# Cosmetic retinoid use in photoaged skin: A review of the compounds, their use and mechanisms of action

**DOI:** 10.1111/ics.13013

**Published:** 2024-08-11

**Authors:** Bezaleel Mambwe, Kieran T. Mellody, Orsolya Kiss, Clare O'Connor, Mike Bell, Rachel E. B. Watson, Abigail K. Langton

**Affiliations:** ^1^ Centre for Dermatology Research Salford Royal NHS Foundation Trust, Manchester Academic Health Science Centre, The University of Manchester Manchester UK; ^2^ No7 Beauty Company, Walgreens Boots Alliance Nottingham UK; ^3^ A*STAR Skin Research Laboratory (A*SRL), Agency for Science, Technology and Research (A*STAR) Singapore City Singapore

**Keywords:** cosmeceuticals, formulation/stability, skin barrier, skin physiology/structure, topical retinoids

## Abstract

The inevitable attrition of skin due to ultraviolet radiation, termed photoaging, can be partially restored by treatment with retinoid compounds. Photoaged skin in lightly pigmented individuals, clinically presents with the appearance of wrinkles, increased laxity, and hyper‐ and hypopigmentation. Underlying these visible signs of ageing are histological features such as epidermal thinning, dermal–epidermal junction flattening, solar elastosis and loss of the dermal fibrillin microfibrillar network, fibrillar collagen and glycosaminoglycans. Retinoid compounds are comprised of three main generations with the first generation (all‐*trans* retinoic acid, retinol, retinaldehyde and retinyl esters) primarily used for the clinical and cosmetic treatment of photoaging, with varying degrees of efficacy, tolerance and stability. All‐*trans* retinoic acid is considered the ‘gold standard’ for skin rejuvenation; however, it is a prescription‐only product largely confined to clinical use. Therefore, retinoid derivatives are readily incorporated into cosmeceutical formulations. The literature reported in this review suggests that retinol, retinyl esters and retinaldehyde that are used in many cosmeceutical products, are efficacious, safe and well‐tolerated. Once in the skin, retinoids utilize a complex signalling pathway that promotes remodelling of photoaged epidermis and dermis and leads to the improvement of the cutaneous signs of photoaging.

## Graphical abstract

This article reviews the cosmetic use of retinoid compounds for improving signs of photoaging. Retinoids, via a complex signalling pathway, can remodel the epidermis and dermis leading to an improved clinical and histological appearance of the skin. (Created with Biorender.com).

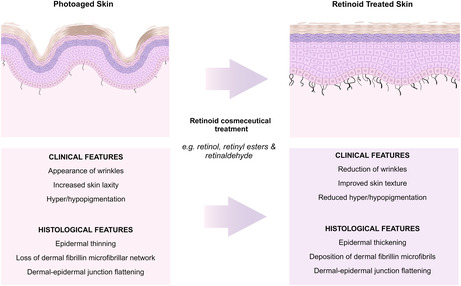

## Résumé

L'altération inévitable de la peau due aux rayons ultraviolets, appelée photovieillissement, peut être partiellement restaurée par un traitement à base de composés rétinoïdes. Chez les personnes à la pigmentation claire, le photovieillissement de la peau se manifeste au plan clinique par l'apparition de rides, un relâchement accru et une hyperpigmentation ou hypopigmentation. Ces signes visibles du vieillissement sont sous‐tendus par des caractéristiques histologiques telles que l'amincissement de l'épiderme, l'aplatissement de la jonction dermo‐épidermique, l'élastose solaire et la perte du réseau microfibrillaire de fibrilline dermique, du collagène fibrillaire et des glycosaminoglycanes. Les composés rétinoïdes sont constitués de trois générations principales, la première génération (acide tout‐*trans* rétinoïque, rétinol, rétinaldéhyde et esters de rétinyle) étant principalement utilisée pour le traitement clinique et cosmétique du photovieillissement, avec des degrés variables d'efficacité, de tolérance et de stabilité. L'acide tout‐*trans* rétinoïque est considéré comme la référence en matière de rajeunissement de la peau; il s'agit toutefois d'un produit délivré uniquement sur ordonnance, dont l’utilisation est largement limitée au domaine clinique. Les dérivés rétinoïdes sont donc volontiers incorporés ds formulations cosméceutiques. La littérature citée dans cette synthèse bibliographique laisse penser que le rétinol, les esters de rétinyle et le rétinaldéhyde, utilisés dans de nombreux produits cosmétiques, sont efficaces, sûrs et bien tolérés. Une fois dans la peau, les rétinoïdes utilisent une voie de signalisation complexe qui favorise le remodelage de l'épiderme et du derme photovieillis, et conduit à l'amélioration des signes cutanés du photovieillissement.

## INTRODUCTION

The skin is the largest organ of the human body and protects the internal organs from external insults [1]. It is a highly complex tissue with multiple cell types and structures that are divided into three anatomical layers: the epidermis (composed mainly of keratinocytes, but also containing antigen‐presenting Langerhans' cells and pigment‐producing melanocytes [2]); the dermis, a complex extracellular matrix (ECM) synthesized by fibroblasts; and the hypodermis, a layer of shock absorbing adipose tissue, a reservoir for pluripotent stem cells [3]. The superficial stratified epithelium of the epidermis is separated from the deeper anatomical layers by a specialized basement membrane, the dermal–epidermal junction (DEJ). Keratinocytes arise in the *stratum basale* from stem cells, that can proliferate almost continuously and subsequently differentiate to form the keratinocyte *strata* of the epithelium. On terminal differentiation, these flattened, enucleated and keratinized cells of the *stratum corneum* function as a physical barrier against pathogens and irritants/toxins [4], and together with tight junctions within the living epidermal cells [5], contribute to the regulation of trans‐epidermal water loss.

## SKIN AGEING

**FIGURE 1:**
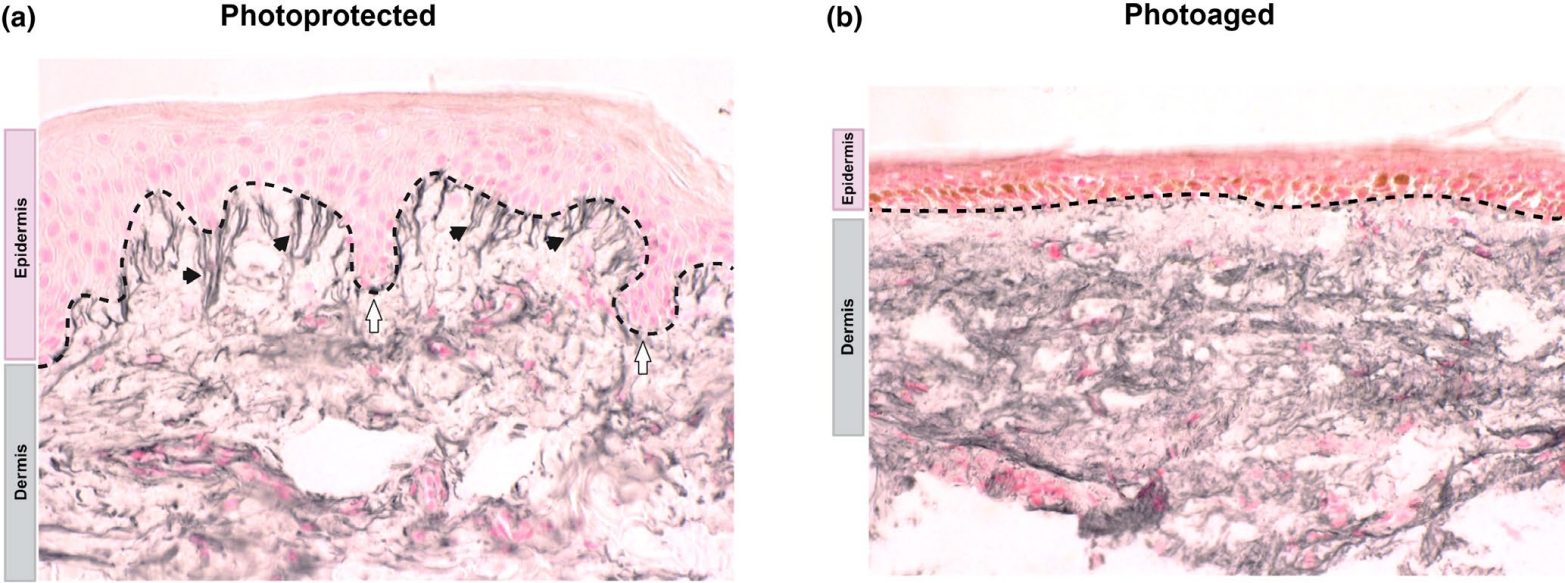
Characteristics of photo‐protected and photoaged skin. Histologically, young photo‐protected skin (a) has a thick epidermis and highly convoluted dermal–epidermal junction with interdigitations into the dermis called rete ridges (white arrows). Dermally, there are fibrillin‐rich microfibrils (black arrows) within the papillary dermis. In contrast, photoaged skin (b) exhibits a thinner epidermis, flattened dermal–epidermal junction (with marked loss of rete ridges) and loss of fibrillin‐rich microfibrils.

The skin undergoes a biological attrition of function and morphology as it ages driven by both intrinsic and extrinsic factors. Intrinsic, or chronological ageing, refers to the internal, genetically driven and natural reduction of skin function usually associated with areas of skin that are protected from ultraviolet radiation (UVR), such as the buttock. Photoaging is defined by the structural and physiological attrition in skin function and morphology in body sites, such as the face or forearm, that are frequently exposed to UVR [6]. In white Northern European populations, chronic photoexposure often results in skin that has deep, coarse wrinkles, increased laxity, and hyper and hypopigmentation [7, 8, 9]. The extensive remodelling underlying these visible signs of ageing can be observed histologically (Figure 1) with epidermal thinning and flattening of rete ridges at the DEJ compromising skin structure and function [10, 11]. The diminishment of the fibrillin‐rich microfibrillar (FRM) network in the papillary dermis is one of the histological hallmarks of photoaged skin that, in part, contributes to the clinical appearance of wrinkles and increased skin laxity [12]. Furthermore, extracellular matrix (ECM) remodelling due to UVR‐mediated ECM breakdown or induction of alternative splicing of the elastin gene leads to inadequate synthesis and deposition of non‐functional elastin by dermal fibroblasts [13] leading to accumulation of disorganized elastotic material or solar elastosis [14, 15, 16, 17]. Extracellular matrix remodelling also involves UVR‐induced crosslinking of collagen fibrils [18, 19], altered collagen glycation, expression and deposition (e.g. collagens type I and VII) [20, 21] and changes to the expression of other key ECM components (e.g. loss of fibulin‐5, loss of glycosaminoglycans) [12, 22, 23, 24]. Together, these changes alter the biophysical properties of the skin and define the photoaged phenotype.

All‐*trans* retinoic acid (ATRA) and derivative compounds have been shown to effectively mitigate cutaneous photoaging when used clinically and cosmetically [25, 26]. This article reviews the family of retinoid compounds and discusses the clinical evidence that supports their cosmeceutical efficacy, together with their proposed mechanism of action.

## RETINOID COMPOUNDS

Retinoids are a class of compounds which are chemically related to vitamin A, a lipophilic hormone comprised of an isoprenoid chain attached to a beta‐ionone ring and occur both naturally and as synthetic derivatives [27]. Vitamin A is required for multiple biological processes such as vision [28], reproduction and embryogenesis [29]; and cellular processes such as proliferation, differentiation and apoptosis [30]. As vitamin A cannot be synthesized by the body, it is an essential dietary vitamin [31]. Vitamin A also occurs naturally as retinyl esters and beta‐carotene which are converted to retinol (ROH) during digestion and back to retinyl esters for storage in the liver [27]. Metabolic processing of ROH within cells yields the biologically active ATRA which can activate nuclear retinoic acid receptors (RARs) and retinoid X receptors (RXRs) [30].

**FIGURE 2:**
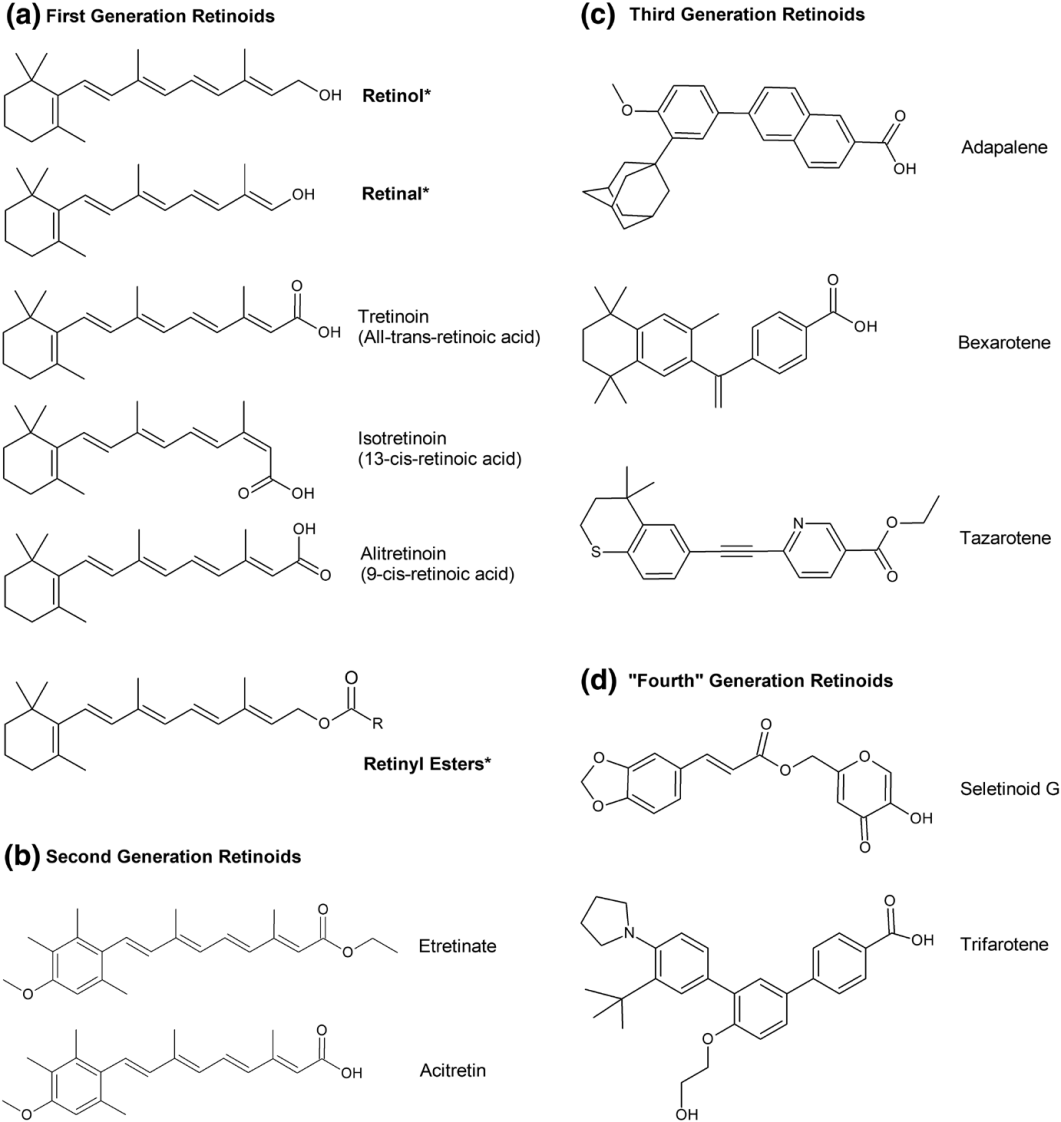
Categorization of retinoid compounds. Characterization of the retinoid family of compounds is based on structure and time of introduction. These are categorized as: Non‐aromatic (a), mono‐aromatic with an N‐terminal benzene ring (b), poly‐aromatic (c) and pyranone‐derived (d). Compounds that have been used in cosmeceutical formulations indicated with an asterisk (*).

The retinoid family are categorized into three main groups, or generations, based on their structure and time of introduction to practice. Naturally occurring retinoids – ATRA (or tretinoin), ROH, retinaldehyde (RAL), 9‐cis‐retinoic acid (alitretinoin) and 13‐cis‐retinoic acid (isotretinoin) – comprise first‐generation, non‐aromatic compounds which also include retinyl esters (Figure 2a). Second‐generation retinoids are mono‐aromatic compounds that possess an N‐terminal benzene ring and include etretinate and acitretin (Figure 2b). Poly‐aromatics, that is, those with multiple aromatic rings, such as tazarotene and adapalene, are examples of third‐generation retinoids (Figure 2c). A more recent generation of retinoid, derived from pyranones, such as seletinoid G and trifarotene, are categorized as the fourth generation (Figure 2d) [25, 32, 33].

Retinoid compounds are routinely used in the treatment of various skin diseases and conditions. In this review, we focus on the use of these compounds in treatment of photoaging.

## RETINOID SIGNALLING PATHWAYS

**FIGURE 3:**
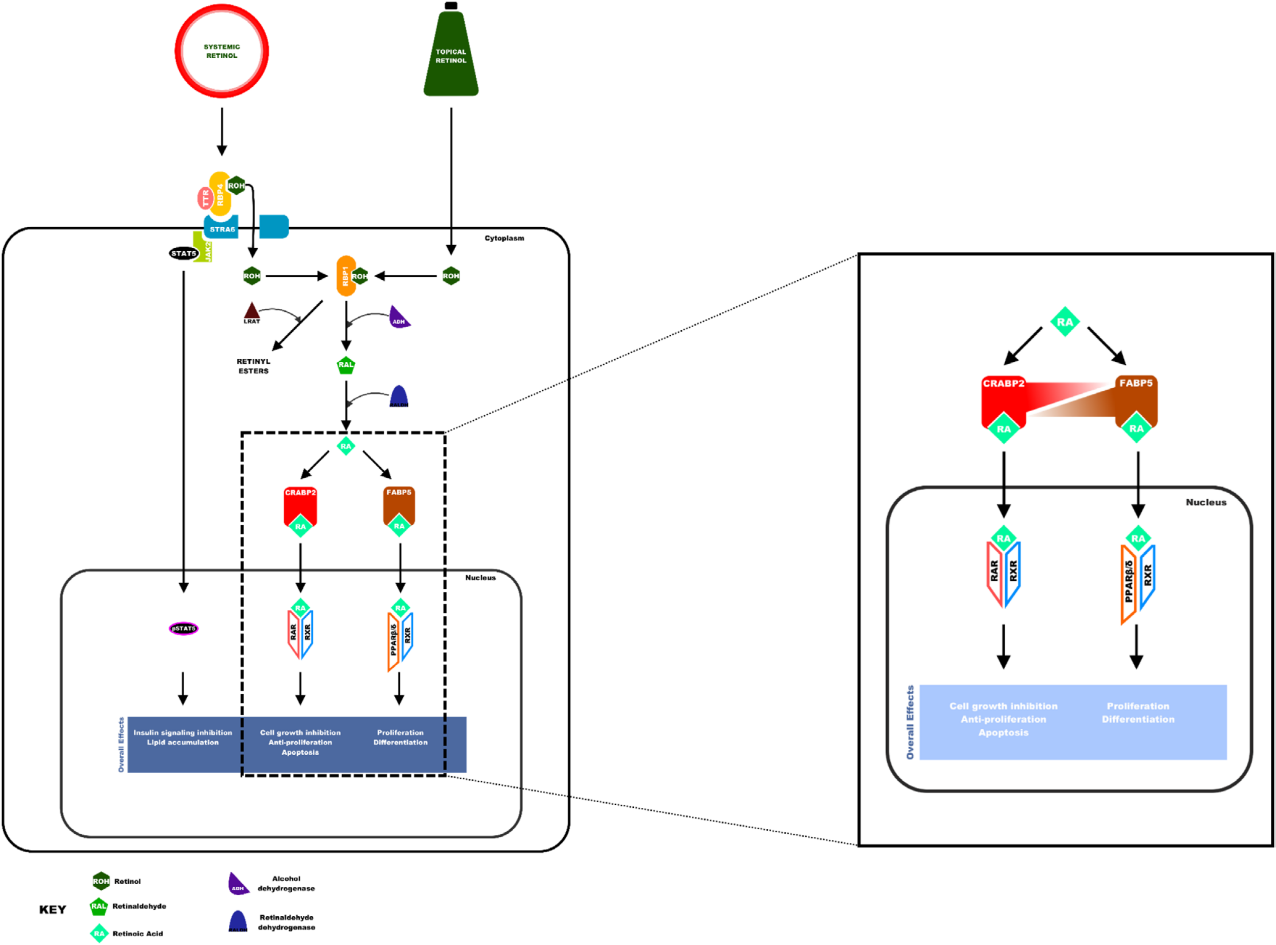
The retinoid signalling pathway. Systemic and topical retinol enters the cell via different pathways but are processed intracellularly in the same way. Processing of retinol to retinoic acid leads to activation of the RAR/RXR or PPARβ/δ/RXR pathways via translocation by CRABP2 or FABP5, respectively. The CRABP2/FABP5 ratio determines which of the nuclear receptors is activated. A higher CRABP2/FABP5 ratio drives RAR/RXR receptor heterodimerization and activation of cell growth arrest and apoptosis. Conversely, a lower CRABP2/FABP5 ratio drives activation of proliferation and differentiation by activating the PPARβ/δ‐RXR pathway.

Retinoid compounds exert their actions by utilizing the intracellular retinoid signalling pathway. It is well established that dietary ROH (from oily fish, fruits and vegetables [34]) relies on the retinol binding protein 4 (RBP4)/stimulated by retinoic acid 6 (STRA6) system to enter and exert its effects in the cell [35]. Once in the cell, cytoplasmic‐free ROH is sequestered by another ROH carrier protein, cellular retinol binding protein 1 (CRBP1). Sequestered ROH can be accessed by the enzyme, lecithin: retinol acyltransferase which preferentially drives the biosynthesis of retinyl esters for storage [36]. Alternatively, ROH can be processed into the active metabolite, ATRA, in a two‐step reaction where alcohol dehydrogenase converts ROH to RAL, then retinaldehyde dehydrogenase converts RAL into ATRA (Figure 3) [37, 38].

Intracellular lipid‐binding proteins (iLBPs) facilitate the translocation of ATRA isoforms into the nucleus. There are multiple iLBP families that bind retinoids, fatty acids and other hydrophobic ligands in a 1:1 complex [37, 39]. Of particular interest are cytoplasmic retinoic acid‐binding protein 2 (CRABP2) and the fatty acid‐binding protein 5 (FABP5), which facilitate translocation of ATRA from the cytoplasm into the nucleus. Nuclear translocation of ATRA via CRABP2 leads to the activation of nuclear RARs. RARs exist in three isoforms in the nucleus, α, β and γ [40] and in the presence of ATRA, RAR isoforms heterodimerize with one of the three RXR isoforms, either α, β or γ. The newly formed RAR/RXR heterodimer with ATRA and co‐activator protein then bind to DNA response elements known as retinoic acid response elements (RAREs) that possess the binding motif A/GGG/TTCA [41]. This leads to transcription of anti‐proliferative genes and cell growth arrest [42, 43]. Conversely, FABP5 delivers ATRA to PPARβ/δ, one of three isoforms of receptors that function as transcription factors by binding to peroxisome proliferator response elements (PPREs) [44]. PPARβ/δ heterodimerization with an RXR isoform takes place and leads to activation of cell survival and proliferation genes such as *FIAF, ADRP* and *PDK‐1* (Figure 3) [45]. The other PPAR isoforms, PPARγ and PPARα, bind fatty acids and eicosanoids thus playing a vital role as lipid sensors and in lipid metabolism regulation [44] but have a very low ATRA binding affinity.

An alternative signalling pathway where STRA6‐mediated activation of the JAK/STAT pathway has also been shown to take place [46]. Binding of the extracellular retinol–protein complex to STRA6 results in the activation of the JAK/STAT pathway by initially recruiting JAK2 to phosphorylate the cytosolic SH2 domain of STRA6. This, in turn, leads to phosphorylation and activation of the transcription factor, STAT5. Phosphorylated STAT5 then translocates to the nucleus to direct STAT5‐mediated transcription of target genes such as *SOCS3* and *PPARγ* [47]. Interestingly, Romana et al. [48] demonstrated that topical retinol attenuated stress‐induced signs of ageing via EGF‐mediated EGFR activation [48], thus suggesting another possible retinoid signalling pathway.

## MECHANISM OF ACTION OF RETINOIDS IN SKIN

Percutaneous penetration of topical ROH formulations is vital to the delivery of active retinoid to skin cells. In the imiquimod‐induced murine model of psoriasis, expression of RBP4 in healthy skin is low, increasing only on induction of psoriasis‐like lesions [49]. This suggested that topical ROH enters the cell via an RBP4‐independent pathway. It should be noted that the RBP4 receptor, STRA6, is expressed in all the layers of the epidermis (mainly cytoplasmic) and in dermal fibroblasts [49]. Therefore, the transport of ROH and other retinoids across the plasma membrane during topical application remains to be clarified, although it has been suggested that retinoids may diffuse across the plasma membrane into the cell; however, facilitating membrane transporters, if any, in the skin remain to be confirmed [50].

There is little known about the intracellular mechanisms involved in processing other topically applied ROH derivatives; yet we can hypothesize that retinyl esters may be first metabolized into ROH by retinyl ester hydrolase and oxidized by alcohol dehydrogenase to form RAL. Retinal dehydrogenase may then act on RAL to produce ATRA [51]. The effect of ATRA in skin is dependent upon cell type and disease/condition. In certain conditions, ATRA has anti‐proliferative and apoptotic effects (i.e., in the treatment of hyperproliferative conditions, such as psoriasis [52]), whilst in others, cell survival and proliferation are enhanced (i.e., in the treatment of age‐associated epidermal atrophy [53]). These opposing effects have been attributed to the epidermal [54, 55] intracellular expression ratio of iLBPs, CRABP2 and FABP5. It has been shown that a high CRABP2/FABP5 ratio leads to activation of anti‐proliferative and pro‐apoptotic transcriptional activity via RAR/RXR (expressed in epidermal keratinocytes and dermal fibroblasts [56, 57]) activation whilst a low CRABP2/FABP5 ratio is linked with proliferative and survival effects via PPARβ/δ activation (Figure 3) [44, 45, 58]. Moreover, treatment of age‐associated epidermal atrophy with retinoic acid or retinol treatment of aged skin also led to the upregulation of anti‐ageing genes, collagen I (COL1A1) and III (COL3A1) [53].

## RETINOIDS IN COSMETIC FORMULATIONS

The first documented use of retinoids for cosmetic purposes came in 1983 when the use of ATRA for the management of mild to moderate facial wrinkles was described, resulting in a reduction of wrinkles and improved skin texture [59]. In 1961, U.S. Society of Cosmetic Chemists founding member, Raymond Reed coined the term “cosmeceutical” to describe a category of products that functioned as both as a ‘cosme'tic and pharma ‘ceutical’ (possessing bioactive activity) [60]. The term was further popularized by Dr Albert Kligman [61] who demonstrated, using electron microscopy, that the use of ATRA could induce changes to the epidermal architecture of facial skin [62]. Subsequent studies reported activation of dermal fibroblasts and de novo synthesis of collagen bundles in the dermis of irradiated nude mice following topical ATRA treatment [63] and a double‐blind, vehicle‐controlled study demonstrated the skin rejuvenating benefits of ATRA on photodamaged skin [64]. These early studies pioneered the way for the modern‐day, widespread topical use of retinoids to improve the appearance of photoaged skin in both a clinical and cosmetic context.

Considered as the ‘gold standard’ in anti‐ageing treatment [65], ATRA is not used in topical cosmeceutical formulations. It is available as prescription‐only due to the mild to moderate skin irritation, such as erythema, peeling and burning, observed after prolonged application [66]. Interestingly, it is these ATRA‐associated skin irritancy issues which may contribute, in part, to the active remodelling of the cutaneous microenvironment that improve the clinical features of photodamaged skin [67, 68, 69].

Therefore, alternative retinoid compounds are used in cosmetic anti‐ageing products delivering similar clinical endpoints without the ATRA‐associated adverse events [70].

ATRA precursors are commonly used in cosmeceutical formulations, due to their better stability and tolerance compared to ATRA [71, 72]. Retinyl esters exhibit greater chemical and photo‐stability, and are better tolerated than ROH or ATRA [73]. However, there is little evidence of the efficacy of individual retinyl esters in improving photoaged skin alone; rather, data suggest that benefits may be seen when used in combination with other compounds such as hydroxy acids [74]. Retinaldehyde is used less commonly in cosmetic products but presents a better safety profile compared to ATRA [75]. However, long‐term and accelerated stability testing of commercial retinoid‐containing cosmetics revealed formulation‐dependent chemical and physical instabilities [76].

## RETINOID TREATMENT OF PHOTOAGING

Retinoid compounds have been shown to result in improvement of the symptoms of photoaged skin. Application of topical ATRA (0.025%–0.1% w/w) to aged facial skin has been demonstrated to reduce fine and coarse wrinkling, hyperpigmentation and improved skin texture [64, 77, 78]. Histologically, ATRA treatment results in compaction of the *stratum corneum* and epidermal thickening, [67, 77, 79] indicative of FABP5‐mediated activation of proliferation and differentiation of the epidermal keratinocytes. As described, the retinoids can elicit both inhibitory (via RARs) or activatory (via PPARβ/δ) effects on keratinocyte survival, proliferation and differentiation dependent upon the CRABP2/FABP5 ratio. In aged skin, the CRABP2/FABP5 ratio is higher due to a decrease in expression of CRABP2 in the epidermis [80]. Therefore, the FABP5‐PPARβ/δ pathway is predominant in aged skin, which is consistent with the epidermal proliferation and increased turnover observed following ATRA treatment. Conversely, ATRA treatment inhibits fibroblast matrix metalloproteinase (MMP)‐1 synthesis in an RAR‐dependent manner [81]. Therefore, the response to ATRA treatment in aged skin is a complex process that involves skin layer‐dependent effects.

**FIGURE 4:**
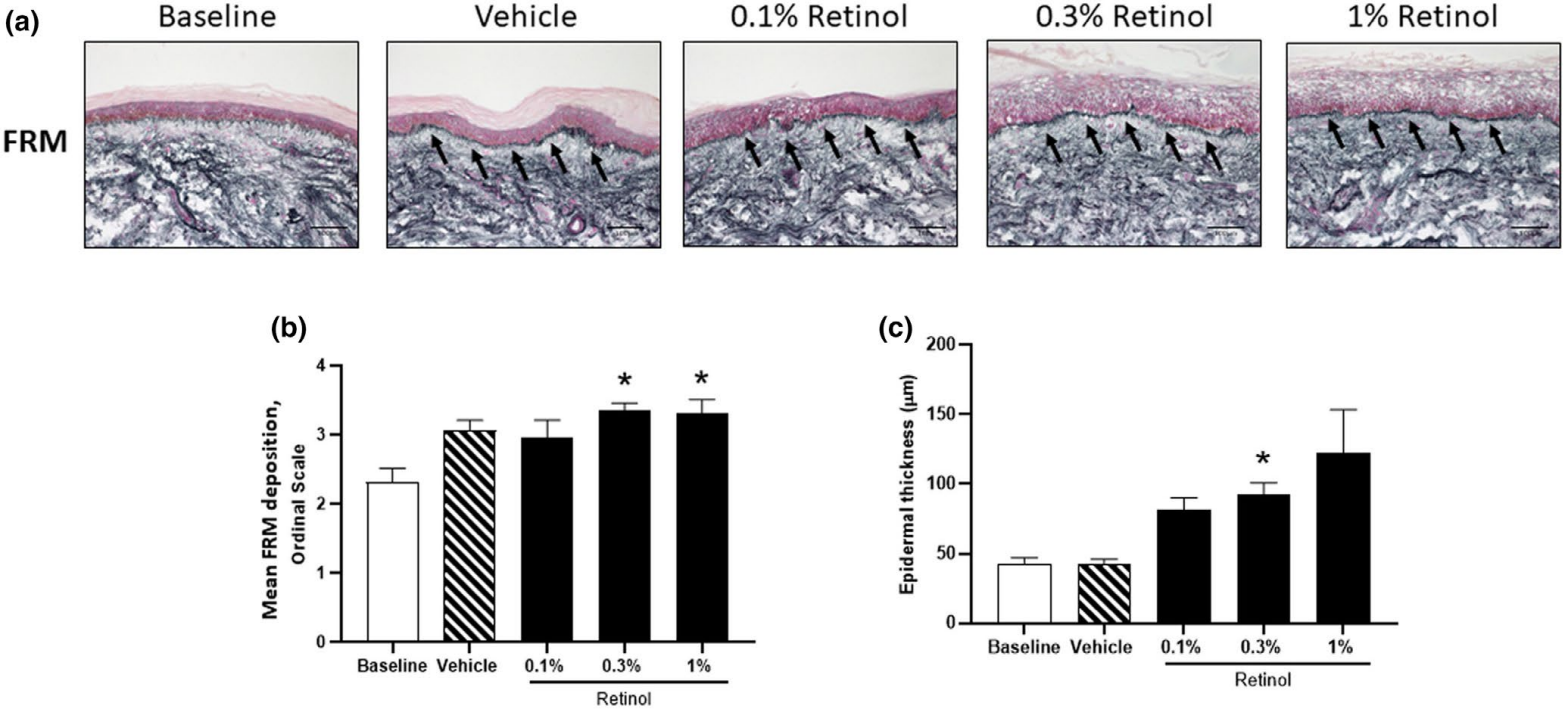
Retinol‐induced increase in epidermal thickness and dermal fibrillin deposition. Retinol induces remodelling by increasing epidermal thickness and the dermal deposition of fibrillin‐rich microfibrils (FRM) in photoaged skin following 12‐day patch test under occlusion. (a) Representative images showing immunostaining for fibrillin‐rich microfibrils. (b) Quantification of FRM deposition by ordinal scoring. (c) Quantification of epidermal thickness. Statistical significance for differences between the treatments compared to the baseline control was assessed by repeated‐measures one‐way ANOVA followed by a Dunnett's multiple comparison test (*p < 0.05). (Extracted from Mellody et al. [86]).

In over‐the‐counter retinoid products, the ROH concentration ranges between 0.05% and 0.3% [82]. Dose‐dependent in vivo studies (starting from 0.1% [83]) show improvement in the appearance of wrinkles, dyspigmentation and skin texture following sustained application [84, 85]. Mellody et al. [86] demonstrated that a 0.3% ROH formulation was equally efficacious in the positive remodelling of dermal and epidermal structure as a 1% formulation (Figure 4), but the lower concentration was better tolerated in northern European skin [86]. Therefore, whilst marginal gains are observed with increasing concentrations, a lower ROH concentration, such as 0.3%, may lessen skin irritancy and improve consumer tolerance.

Retinaldehyde has been explored as a potential photoaging treatment but to a lesser extent than ROH. In the published clinical studies available, a concentration of 0.05% (w/w) RAL improved photoaged skin by increasing epidermal thickness and cutaneous elasticity as assessed by ultrasound and rheological techniques following 1‐year of application [87]. Furthermore, 0.1% RAL applied daily for 8 weeks was better tolerated and as effective as glycolic acid chemical peels, in reducing wrinkles and improving skin texture of photoaged skin [88]. A randomized double‐blind study of a female Korean cohort found that both 0.05% and 0.1% RAL improved overall photoaging by reducing wrinkles, transepidermal water loss and increasing skin hydration. Interestingly, there was no significant difference between the two concentrations other than 0.1% RAL improving the patients' melanin index [75].

Retinyl retinoate is a synthetic retinyl ester created by a condensation reaction between ROH and ATRA. This retinyl ester exhibits better photo‐ and thermal stability compared to ROH and has equal bioactivity to ATRA, as shown by in vitro deposition of collagen following treatment [89]. Retinyl retinoate (0.06%) applied twice daily for 8‐ or 12‐weeks significantly improved periorbital wrinkles in Korean women (>30 years old) compared to ROH (0.075%) or placebo and severe side effects were not observed [90]. In an early study, Green et al. [74] found that 0.15% retinyl propionate induced no significant differences compared to placebo in the clinical and histological features of photoaged skin in 75 subjects following 24 weeks of application, whilst Fu et al. [91] found that 0.3% retinyl propionate in formulation with niacinamide and peptides, reduced wrinkles after 8 weeks. At 24 weeks, this formulation similarly improved signs of photoaging but was better tolerated than ATRA [91]. However, this may have been due to the effects of the other active ingredients (i.e. niacinamide [92] and peptides; See ref. [93]).

Retinyl propionate (0.37%) in combination with climbazole, a topical antifungal, was better tolerated and more efficacious than 0.1% retinol, showing improvement in deep wrinkles in 45 healthy Caucasian females following a 16‐week facial study [94]. In another study, retinyl propionate and hydroxypinacolone retinoate (a cosmetic‐grade retinyl ester) synergistically improved skin ageing in Chinese subjects by improving wrinkles, transepidermal water loss, skin elasticity and smoothness. Expectedly, this combination elicited similar efficacy to ROH without the adverse effects [95]. In a study of Asian males (mean age of 25.5 years), application of two commercially available creams containing retinyl palmitate in a split‐face study showed significant improvements in skin smoothness and wrinkling after 60 days [96]. Similarly, Rawlings et al. [97] found that in cohort of 25 female subjects comprising of multiple ethnicities, an oil‐based ATRA palmitate improved fine lines, wrinkles, pigmentation and overall skin texture over 12‐weeks [97]. Watson et al. [98] assessed the efficacy of a commercially available cosmetic anti‐ageing product containing retinyl palmitate, peptides and antioxidants. Histological analysis in nine photoaged subjects following a 12‐day patch test showed significant deposition of FRM and procollagen I in the papillary dermis [98].

In studies where a retinoid is used in formulation with other actives [91, 94, 97, 98], care should be taken when interpreting the benefits as solely retinoid mediated. In fact, it is possible that the benefits observed could have been due to a single ingredient (e.g., peptides) or a synergistic combination of ingredients.

### Retinoid‐induced epidermal changes

Whilst the mechanism of how topically applied retinoids penetrate the *stratum corneum* to induce their activity is yet to be fully elucidated, these compounds act upon the epidermis to stimulate basal keratinocyte proliferation resulting in epidermal hyperplasia [99, 100]. Although retinoids are anti‐inflammatory compounds [101], their topical application often causes skin irritancy termed retinoid‐induced dermatitis. The dryness and scaling of retinoid‐induced dermatitis is associated with changes to the expression of cornified envelope proteins (e.g., loricirin and small‐proline‐rich proteins) [68], loss of corneocyte cohesion [102], hyperproliferation of the basal keratinocytes [103] and accelerated desquamation [104]. These physiological changes in response to ATRA and other retinoids, alter the functional integrity of the epidermal barrier resulting in increased trans‐epidermal water loss [62, 105, 106, 107]. Retinoid‐induced dermatitis is often short‐lived although some reports of intermittent irritancy have been associated with continued use [108]. The overall integrity of the skin's barrier eventually improves, and the use of emollients can help to counter irritancy problems [109, 110].

Thickening of the *stratum corneum* is concomitant with a reduction in expression of the cell‐to‐cell interaction proteins forming the desmosomal and tight junctions [102, 111]. In addition, topical ROH (1% or 0.3%) reduces E‐cadherin expression throughout the epidermis [86] and this may expedite keratinocyte motility through the epidermal *strata* towards their terminal‐differentiation fate [112]. Recent data in black skin suggest both ATRA (0.025%) and ROH (1% and 0.3%) mediate epidermal remodelling through increased keratinocyte proliferation (increased Ki67 expression) [113] in a manner similar to that previously observed in white skin [86].

Retinoids used alone [114, 115, 116, 117, 118], or in combination with other actives such as hydroquinone [119, 120, 121, 122], reduce hyperpigmentation in photoaged skin. However, the mechanism is unclear since a direct inhibitory effect upon melanocytes and melanogenesis has not been fully established [123]. Conflicting data have been reported in the irradiated skin of mice treated with retinoids [124]; their ability to reduce hyperpigmentation may be driven by indirect mechanisms such as keratinocyte proliferation. The rapid turnover of keratinocytes accelerates their movement through epidermal *strata* and may limit the time available to allow acquisition of melanin deposits within keratinocytes. This may explain why reduced seasonal melanin ascent to the supra‐basal layers in response to retinol has been reported [125].

Hyaluronic acid (HA) is the main glycosaminoglycan present in skin and is known for its water‐holding capacity [126]. HA levels in the skin have been shown to decrease with age and result in loss of skin moisture in turn affecting skin firmness [127]. In a 52‐week double blind clinical study of aged skin, Li et al [128] showed an increase in hyaluronan synthase enzyme expression which significantly increased the epidermal production of HA following treatment with a stabilized retinol formulation. This further showed the multifaceted benefits of retinol treatment in aged skin.

### Retinoid‐induced remodelling of the dermal ECM

Retinoid‐induced remodelling of the dermal ECM may also occur in conjunction with epidermal changes. This is particularly evident in aged or photoaged skin, where ECM synthesis (deposition of fibrillar collagens and FRM) is reduced. Dermal fibroblasts are the main cell type responsible for homeostatic remodelling the dermal ECM. However, their inability to metabolize systemic ATRA but not ROH [129], suggests that keratinocytes are the key player responsible for the observed responses following topical application of retinoids. Multiple studies have used ATRA (4‐day occlusion) and ROH (12‐day occlusion) in skin patch tests and have shown that the papillary dermal FRM network is replenished by treatment [69]. The propensity of keratinocytes to express fibrillin [12, 130] and the lack of dermal fibroblasts proximal to the DEJ suggests basal keratinocytes are likely responsible for inducing the de novo synthesis and deposition of the papillary dermal FRM network, induced by topical retinoid treatments [12].

Retinoids have been shown to activate the synthesis of new collagen by dermal fibroblasts [99, 100, 131]. Simultaneously, MMP activity is reduced and that of tissue inhibitors of matrix metalloproteinases increased [132, 133]. Interestingly, a recent double‐blind comparative study found that ROH augmented collagen deposition above that seen with ATRA [134], suggesting differences in their pharmacodynamics. Furthermore, retinol can improve skin hydration by upregulating glycosaminoglycan production [135].

## CONCLUSION AND FUTURE PERSPECTIVES

Exposure to UVR is inevitable but measures such as the use of topical ATRA and its precursors can be successfully used to normalize skin function and improve the clinical appearance of photoaged skin. Retinoid signalling is a complex process and whilst much is known about the overall mechanisms involved, specific details of how retinoids exert their pluripotent actions remain unclear. However, the literature suggests that the CRABP2/FABP5 ratio is critical in determining the effect of retinoid treatment. Furthermore, retinoids exert opposing effects on cell proliferation; with pro‐proliferative effects observed when treating photoaged skin yet anti‐proliferative effects are observed for psoriasis treatment [136].

Although this review has focussed on the positive changes afforded to skin following retinoid treatment, striking a balance between tolerability, safety & efficacy is an ongoing challenge. Identifying the optimal retinoid dose where significant efficacy is achieved combined with the least tolerance issues continues to be investigated. This is especially important in skin of colour where intolerance has a greater risk of inducing post inflammatory hyperpigmentation (PIH) [137]. Nevertheless, the development of new technologies that facilitate their chemical and photo‐stability, and percutaneous penetration, promises greater opportunity for the continued use of retinoids to rejuvenate skin in both pharmaceutical and cosmeceutical formulations.

Whilst this review identified some literature describing the use of retinoids in Asian and black skin, there remains scope of further investigation of the benefits and risks of retinoid use in more diverse skin types. For example, chronically photo‐exposed skin of colour (Fitzpatrick phototypes V–VI) is biomechanically characterized by loss of elasticity and firmness and histologically by the thinning of the epidermis, loss of rete ridges and dermal matrix proteins, for example, FRM and fibulin‐5 [138]. These are hallmarks of photoaging that are routinely treated with retinoids in lightly pigmented skin (Fitzpatrick phototypes I–III). Therefore, determining the optimal concentrations, assessing tolerance and determining best practices of assessing efficacy for each ATRA precursor is important to address photoaging in skin of colour. In conclusion, topical retinoids are an effective, accessible and a relatively low‐risk rejuvenation strategy for the improvement of skin photoaging.

## CONFLICT OF INTEREST STATEMENT

The authors declare no known conflicts of interest.

## References

[1] Boer M , Duchnik E , Maleszka R , Marchlewicz M . Structural and biophysical characteristics of human skin in maintaining proper epidermal barrier function. Postepy Dermatol Alergol. 2016;33(1):1–5. 26985171 10.5114/pdia.2015.48037 PMC4793052

[2] Madison KC . Barrier function of the skin: "la raison d'être" of the epidermis. J Invest Dermatol. 2003;121(2):231–241. 12880413 10.1046/j.1523-1747.2003.12359.x

[3] Arda O , Göksügür N , Tüzün Y . Basic histological structure and functions of facial skin. Clin Dermatol. 2014;32(1):3–13. 24314373 10.1016/j.clindermatol.2013.05.021

[4] Fore J . A review of skin and the effects of aging on skin structure and function. Ostomy Wound Manage. 2006;52(9):24–35; quiz 6–7. 16980727

[5] Svoboda M , Bílková Z , Muthný T . Could tight junctions regulate the barrier function of the aged skin? J Dermatol Sci. 2016;81(3):147–152. 26639794 10.1016/j.jdermsci.2015.11.009

[6] Farage MA , Miller KW , Elsner P , Maibach HI . Intrinsic and extrinsic factors in skin ageing: a review. Int J Cosmet Sci. 2008;30(2):87–95. 18377617 10.1111/j.1468-2494.2007.00415.x

[7] Langton AK , Ali Z , Hann M , Ayer J , Watson REB , Griffiths CEM . Prevalence of atrophic and hypertrophic skin ageing phenotypes: a UK‐based observational study. Acta Derm Venereol. 2020;100(19):adv00347. 33241421 10.2340/00015555-3708 PMC9309709

[8] Langton AK , Ayer J , Griffiths TW , Rashdan E , Naidoo K , Caley MP , et al. Distinctive clinical and histological characteristics of atrophic and hypertrophic facial photoageing. J Eur Acad Dermatol Venereol. 2021;35(3):762–768. 33275818 10.1111/jdv.17063 PMC7986784

[9] Sachs DL , Varani J , Chubb H , Fligiel SEG , Cui Y , Calderone K , et al. Atrophic and hypertrophic photoaging: clinical, histologic, and molecular features of 2 distinct phenotypes of photoaged skin. J Am Acad Dermatol. 2019;81(2):480–488. 30954583 10.1016/j.jaad.2019.03.081

[10] Boss GR , Seegmiller JE . Age‐related physiological changes and their clinical significance. West J Med. 1981;135(6):434–440. 7336713 PMC1273316

[11] Langton AK , Graham HK , Griffiths CEM , Watson REB . Ageing significantly impacts the biomechanical function and structural composition of skin. Exp Dermatol. 2019;28(8):981–984. 31152614 10.1111/exd.13980 PMC6851988

[12] Watson RE , Griffiths CE , Craven NM , Shuttleworth CA , Kielty CM . Fibrillin‐rich microfibrils are reduced in photoaged skin. Distribution at the dermal‐epidermal junction. J Invest Dermatol. 1999;112(5):782–787. 10233772 10.1046/j.1523-1747.1999.00562.x

[13] Weihermann AC , Lorencini M , Brohem CA , de Carvalho CM . Elastin structure and its involvement in skin photoageing. Int J Cosmet Sci. 2017;39(3):241–247. 27731897 10.1111/ics.12372

[14] Mitchell RE . Chronic solar dermatosis: a light and electron microscopic study of the dermis. J Invest Dermatol. 1967;48(3):203–220. 6020685 10.1038/jid.1967.33

[15] Ma W , Wlaschek M , Tantcheva‐Poor I , Schneider LA , Naderi L , Razi‐Wolf Z , et al. Chronological ageing and photoageing of the fibroblasts and the dermal connective tissue. Clin Exp Dermatol. 2001;26(7):592–599. 11696063 10.1046/j.1365-2230.2001.00905.x

[16] Bilac C , Sahin MT , Ozturkcan S . Chronic actinic damage of facial skin. Clin Dermatol. 2014;32(6):752–762. 25441468 10.1016/j.clindermatol.2014.02.014

[17] Uitto J . The role of elastin and collagen in cutaneous aging: intrinsic aging versus photoexposure. J Drugs Dermatol. 2008;7(2 Suppl):s12–s16. 18404866

[18] Avery NC , Bailey AJ . The effects of the Maillard reaction on the physical properties and cell interactions of collagen. Pathol Biol (Paris). 2006;54(7):387–395. 16962252 10.1016/j.patbio.2006.07.005

[19] Dalle Carbonare M , Pathak MA . Skin photosensitizing agents and the role of reactive oxygen species in photoaging. J Photochem Photobiol B. 1992;14(1–2):105–124. 1331386 10.1016/1011-1344(92)85086-a

[20] Craven NM , Watson RE , Jones CJ , Shuttleworth CA , Kielty CM , Griffiths CE . Clinical features of photodamaged human skin are associated with a reduction in collagen VII. Br J Dermatol. 1997;137(3):344–350. 9349327

[21] Griffiths C , Russman AN , Majmudar G , Singer RS , Hamilton TA , Voorhees JJ . Restoration of collagen formation in photodamaged human skin by tretinoin (retinoic acid). N Engl J Med. 1993;329(8):530–535. 8336752 10.1056/NEJM199308193290803

[22] Kadoya K , Sasaki T , Kostka G , Timpl R , Matsuzaki K , Kumagai N , et al. Fibulin‐5 deposition in human skin: decrease with ageing and ultraviolet B exposure and increase in solar elastosis. Br J Dermatol. 2005;153(3):607–612. 16120151 10.1111/j.1365-2133.2005.06716.x

[23] Varani J , Dame MK , Rittie L , Fligiel SE , Kang S , Fisher GJ , et al. Decreased collagen production in chronologically aged skin: roles of age‐dependent alteration in fibroblast function and defective mechanical stimulation. Am J Pathol. 2006;168(6):1861–1868. 16723701 10.2353/ajpath.2006.051302 PMC1606623

[24] Oikarinen A . The aging of skin: chronoaging versus photoaging. Photodermatol Photoimmunol Photomed. 1990;7(1):3–4. 2371168

[25] Mukherjee S , Date A , Patravale V , Korting HC , Roeder A , Weindl G . Retinoids in the treatment of skin aging: an overview of clinical efficacy and safety. Clin Interv Aging. 2006;1(4):327–348. 18046911 10.2147/ciia.2006.1.4.327 PMC2699641

[26] Zasada M , Budzisz E . Retinoids: active molecules influencing skin structure formation in cosmetic and dermatological treatments. Postepy Dermatol Alergol. 2019;36(4):392–397. 31616211 10.5114/ada.2019.87443 PMC6791161

[27] Blaner WS , Li Y , Brun PJ , Yuen JJ , Lee SA , Clugston RD . Vitamin A absorption, storage and mobilization. Subcell Biochem. 2016;81:95–125. 27830502 10.1007/978-94-024-0945-1_4

[28] Saari JC . Vitamin A and vision. Subcell Biochem. 2016;81:231–259. 27830507 10.1007/978-94-024-0945-1_9

[29] Clagett‐Dame M , Knutson D . Vitamin A in reproduction and development. Nutrients. 2011;3(4):385–428. 22254103 10.3390/nu3040385 PMC3257687

[30] Roos TC , Jugert FK , Merk HF , Bickers DR . Retinoid metabolism in the skin. Pharmacol Rev. 1998;50(2):315–333. 9647871

[31] Antille C , Tran C , Sorg O , Saurat JH . Penetration and metabolism of topical retinoids in ex vivo organ‐cultured full‐thickness human skin explants. Skin Pharmacol Physiol. 2004;17(3):124–128. 15087591 10.1159/000077238

[32] Riahi RR , Bush AE , Cohen PR . Topical retinoids: therapeutic mechanisms in the treatment of photodamaged skin. Am J Clin Dermatol. 2016;17(3):265–276. 26969582 10.1007/s40257-016-0185-5

[33] Naik PP . Trifarotene: a novel therapeutic option for acne. Dermatol Res Pract. 2022;2022:1504303. 35668721 10.1155/2022/1504303 PMC9166940

[34] Lerner UH , Vitamin A . Discovery, metabolism, receptor signaling and effects on bone mass and fracture susceptibility. Front Endocrinol (Lausanne). 2024;15:1298851. 38711977 10.3389/fendo.2024.1298851 PMC11070503

[35] Blomhoff R , Green MH , Berg T , Norum KR . Transport and storage of vitamin A. Science. 1990;250(4979):399–404. 2218545 10.1126/science.2218545

[36] Ruiz A , Winston A , Lim Y‐H , Gilbert BA , Rando RR , Bok D . Molecular and biochemical characterization of lecithin retinol acyltransferase. J Biol Chem. 1999;274(6):3834–3841. 9920938 10.1074/jbc.274.6.3834

[37] Napoli JL . Functions of intracellular retinoid binding‐proteins. Subcell Biochem. 2016;81:21–76. 27830500 10.1007/978-94-024-0945-1_2 PMC5493979

[38] Bliss AF . The equilibrium between vitamin a alcohol and aldehyde in the presence of alcohol dehydrogenase. Arch Biochem Biophys. 1951;31(2):197–204. 14830226 10.1016/0003-9861(51)90206-8

[39] Bernlohr DA , Simpson MA , Hertzel AV , Banaszak LJ . Intracellular lipid‐binding proteins and their genes. Annu Rev Nutr. 1997;17:277–303. 9240929 10.1146/annurev.nutr.17.1.277

[40] Chambon P . A decade of molecular biology of retinoic acid receptors. FASEB J. 1996;10(9):940–954. 8801176

[41] Umesono K , Murakami KK , Thompson CC , Evans RM . Direct repeats as selective response elements for the thyroid hormone, retinoic acid, and vitamin D3 receptors. Cell. 1991;65(7):1255–1266. 1648450 10.1016/0092-8674(91)90020-y PMC6159884

[42] Vreeland AC , Levi L , Zhang W , Berry DC , Noy N . Cellular retinoic acid‐binding protein 2 inhibits tumor growth by two distinct mechanisms. J Biol Chem. 2014;289(49):34065–34073. 25320093 10.1074/jbc.M114.604041 PMC4256341

[43] Feng X , Zhang M , Wang B , Zhou C , Mu Y , Li J , et al. CRABP2 regulates invasion and metastasis of breast cancer through hippo pathway dependent on ER status. J Exp Clin Cancer Res. 2019;38(1):361. 31419991 10.1186/s13046-019-1345-2 PMC6697986

[44] Berger J , Moller DE . The mechanisms of action of PPARs. Annu Rev Med. 2002;53:409–435. 11818483 10.1146/annurev.med.53.082901.104018

[45] Schug TT , Berry DC , Shaw NS , Travis SN , Noy N . Opposing effects of retinoic acid on cell growth result from alternate activation of two different nuclear receptors. Cell. 2007;129(4):723–733. 17512406 10.1016/j.cell.2007.02.050 PMC1948722

[46] Gliniak CM , Brown JM , Noy N . The retinol‐binding protein receptor STRA6 regulates diurnal insulin responses. J Biol Chem. 2017;292(36):15080–15093. 28733465 10.1074/jbc.M117.782334 PMC5592683

[47] Berry DC , Jin H , Majumdar A , Noy N . Signaling by vitamin A and retinol‐binding protein regulates gene expression to inhibit insulin responses. Proc Natl Acad Sci USA. 2011;108(11):4340–4345. 21368206 10.1073/pnas.1011115108 PMC3060239

[48] Romana‐Souza B , Silva‐Xavier W , Monte‐Alto‐Costa A . Topical retinol attenuates stress‐induced ageing signs in human skin ex vivo, through EGFR activation via EGF, but not ERK and AP‐1 activation. Exp Dermatol. 2019;28(8):906–913. 29704879 10.1111/exd.13675

[49] Wang H‐M , Wu C , Jiang Y‐Y , Wang W‐M , Jin H‐Z . Retinol and vitamin a metabolites accumulate through RBP4 and STRA6 changes in a psoriasis murine model. Nutr Metab (Lond). 2020;17:5. 31956331 10.1186/s12986-019-0423-y PMC6958599

[50] Sun H , Kawaguchi R . The membrane receptor for plasma retinol‐binding protein, a new type of cell‐surface receptor. Int Rev Cell Mol Biol. 2011;288:1–41. 21482409 10.1016/B978-0-12-386041-5.00001-7 PMC3907177

[51] D'Ambrosio DN , Clugston RD , Blaner WS . Vitamin A metabolism: an update. Nutrients. 2011;3(1):63–103. 21350678 10.3390/nu3010063 PMC3042718

[52] Psomadakis CE , Han G . New and emerging topical therapies for psoriasis and atopic dermatitis. J Clin Aesthet Dermatol. 2019;12(12):28–34. PMC7002051 32038762

[53] Kong R , Cui Y , Fisher GJ , Wang X , Chen Y , Schneider LM , et al. A comparative study of the effects of retinol and retinoic acid on histological, molecular, and clinical properties of human skin. J Cosmet Dermatol. 2016;15(1):49–57. 26578346 10.1111/jocd.12193

[54] Siegenthaler G , Hotz R , Chatellardgruaz D , Jaconi S , Saurat JH . Characterization and expression of a novel human fatty acid‐binding protein: the epidermal type (E‐FABP). Biochem Biophys Res Commun. 1993;190(2):482–487. 8427590 10.1006/bbrc.1993.1073

[55] Eller MS , Harkness DD , Jag B , Gilchrest BA . Epidermal differentiation enhances CRABP II expression in human skin. J Invest Dermatol. 1994;103(6):785–790. 7798615 10.1111/1523-1747.ep12413037

[56] Reichrath J , Mittmann M , Kamradt J , Müller SM . Expression of retinoid‐X receptors (−alpha,‐beta,‐gamma) and retinoic acid receptors (−alpha,‐beta,‐gamma) in normal human skin: an immunohistological evaluation. Histochem J. 1997;29(2):127–133. 9147069 10.1023/a:1026481205135

[57] Redfern CPF , Todd C . Retinoic acid receptor expression in human skin keratinocytes and dermal fibroblasts in vitro. J Cell Sci. 1992;102(1):113–121. 1323569 10.1242/jcs.102.1.113

[58] Napoli JL . Cellular retinoid binding‐proteins, CRBP, CRABP, FABP5: effects on retinoid metabolism, function and related diseases. Pharmacol Ther. 2017;173:19–33. 28132904 10.1016/j.pharmthera.2017.01.004 PMC5408321

[59] Cordero A . La vitamina A acida en la piel senil. Actua Ter Dermatol. 1983;6:49–54.

[60] Singh NS . Evolution of cosmeceutical: a summary of journey from general cosmetic to cure skin disease. Mod Appl Pharm Pharmacol. 2023;3(1):1–3.

[61] Kligman A . The future of cosmeceuticals: an interview with Albert Kligman, MD, PhD. Interview by Zoe Diana Draelos. Dermatol Surg. 2005;31(7 Pt 2):890–891. 16029684

[62] Kligman AM , Grove GL , Hirose R , Leyden JJ . Topical tretinoin for photoaged skin. J Am Acad Dermatol. 1986;15(4 Pt 2):836–859. 3771853 10.1016/s0190-9622(86)70242-9

[63] Kligman LH . Effects of all‐trans‐retinoic acid on the dermis of hairless mice. J Am Acad Dermatol. 1986;15(4 Pt 2):779–785, 884–7. 3771852 10.1016/s0190-9622(86)70234-x

[64] Weiss JS , Ellis CN , Headington JT , Tincoff T , Hamilton TA , Voorhees JJ . Topical tretinoin improves photoaged skin. A double‐blind vehicle‐controlled study. JAMA. 1988;259(4):527–532. 3336176

[65] Milosheska D , Roškar R . Use of retinoids in topical antiaging treatments: a focused review of clinical evidence for conventional and nanoformulations. Adv Ther. 2022;39(12):5351–5375. 36220974 10.1007/s12325-022-02319-7 PMC9618501

[66] Olsen EA , Irving Katz H , Levine N , Shupack J , Billys MM , Prawer S , et al. Tretinoin emollient cream: a new therapy for photodamaged skin. J Am Acad Dermatol. 1992;26(2, Part 1):215–224. 1552056 10.1016/0190-9622(92)70030-j

[67] Griffiths CE , Finkel LJ , Tranfaglia MG , Hamilton TA , Voorhees JJ . An in vivo experimental model for effects of topical retinoic acid in human skin. Br J Dermatol. 1993;129(4):389–394. 8217750 10.1111/j.1365-2133.1993.tb03163.x

[68] Cheong KA , Kim HJ , Kim JY , Kim CH , Lim WS , Noh M , et al. Retinoic acid and hydroquinone induce inverse expression patterns on cornified envelope‐associated proteins: implication in skin irritation. J Dermatol Sci. 2014;76(2):112–119. 25240866 10.1016/j.jdermsci.2014.08.003

[69] Watson REB , Craven NM , Kang SW , Jones CJP , Kielty CM , Griffiths CEM . A short‐term screening protocol, using fibrillin‐1 as a reporter molecule, for photoaging repair agents. J Invest Dermatol. 2001;116(5):672–678. 11348454 10.1046/j.1523-1747.2001.01322.x

[70] Griffiths TW , Watson REB , Langton AK . Skin ageing and topical rejuvenation strategies. Br J Dermatol. 2023;189(Supplement_1):i17–i23. 37903073 10.1093/bjd/ljad282

[71] Fluhr JW , Vienne MP , Lauze C , Dupuy P , Gehring W , Gloor M . Tolerance profile of retinol, retinaldehyde and retinoic acid under maximized and long‐term clinical conditions. Dermatology. 1999;199(Suppl. 1):57–60. 10.1159/000051381 10473963

[72] Spierings NMK . Evidence for the efficacy of over‐the‐counter vitamin A cosmetic products in the improvement of facial skin aging: a systematic review. J Clin Aesthet Dermatol. 2021;14(9):33–40. 34980969 PMC8675340

[73] O'Byrne SM , Blaner WS . Retinol and retinyl esters: biochemistry and physiology. J Lipid Res. 2013;54(7):1731–1743. 23625372 10.1194/jlr.R037648 PMC3679378

[74] Green C , Orchard G , Cerio R , Hawk JL . A clinicopathological study of the effects of topical retinyl propionate cream in skin photoageing. Clin Exp Dermatol. 1998;23(4):162–167. 9894360 10.1046/j.1365-2230.1998.00331.x

[75] Kwon HS , Lee JH , Kim GM , Bae JM . Efficacy and safety of retinaldehyde 0.1% and 0.05% creams used to treat photoaged skin: a randomized double‐blind controlled trial. J Cosmet Dermatol. 2018;17(3):471–476. 29663701 10.1111/jocd.12551

[76] Temova Rakuša Ž , Škufca P , Kristl A , Roškar R . Retinoid stability and degradation kinetics in commercial cosmetic products. J Cosmet Dermatol. 2021;20(7):2350–2358. 33206444 10.1111/jocd.13852

[77] Lever L , Kumar P , Marks R . Topical retinoic acid for treatment of solar damage. Br J Dermatol. 1990;122(1):91–98. 2404514 10.1111/j.1365-2133.1990.tb08244.x

[78] Griffiths CE , Kang S , Ellis CN , Kim KJ , Finkel LJ , Ortiz‐Ferrer LC , et al. Two concentrations of topical tretinoin (retinoic acid) cause similar improvement of photoaging but different degrees of irritation. A double‐blind, vehicle‐controlled comparison of 0.1% and 0.025% tretinoin creams. Arch Dermatol. 1995;131(9):1037–1044. 7544967

[79] Weinstein GD , Nigra TP , Pochi PE , Savin RC , Allan A , Benik K , et al. Topical tretinoin for treatment of photodamaged skin. A multicenter study. Arch Dermatol. 1991;127(5):659–665. 2024983

[80] Bielli A , Scioli MG , D'Amico F , Tarquini C , Agostinelli S , Costanza G , et al. Cellular retinoic acid binding protein‐II expression and its potential role in skin aging. Aging (Albany NY). 2019;11(6):1619–1632. 30888968 10.18632/aging.101813 PMC6461173

[81] Pan L , Chamberlain SH , Auble DT , Brinckerhoff CE . Differential regulation of collagenase gene expression by retinoic acid receptors – alpha, beta and gamma. Nucleic Acids Res. 1992;20(12):3105–3111. 1320254 10.1093/nar/20.12.3105 PMC312445

[82] Rousselle C . Opinion of the Scientific Committee on Consumer Safety (SCCS) – final version of the opinion on vitamin A (retinol, retinyl acetate and retinyl palmitate) in cosmetic products. Regul Toxicol Pharmacol. 2017;84:102–104. 27856323 10.1016/j.yrtph.2016.11.017

[83] Tucker‐Samaras S , Zedayko T , Cole C , Miller D , Wallo W , Leyden JJ . A stabilized 0.1% retinol facial moisturizer improves the appearance of photodamaged skin in an eight‐week, double‐blind, vehicle‐controlled study. J Drugs Dermatol. 2009;8(10):932–936. 19852122

[84] Zasada M , Budzisz E , Erkiert‐Polguj A . A clinical anti‐ageing comparative study of 0.3 and 0.5% retinol serums: a clinically controlled trial. Skin Pharmacol Physiol. 2020;33(2):102–116. 32428912 10.1159/000508168

[85] Shao Y , He T , Fisher GJ , Voorhees JJ , Quan T . Molecular basis of retinol anti‐ageing properties in naturally aged human skin in vivo. Int J Cosmet Sci. 2017;39(1):56–65. 27261203 10.1111/ics.12348 PMC5136519

[86] Mellody KT , Bradley EJ , Mambwe B , Cotterell Lindsay F , Kiss O , Halai P , et al. Multifaceted amelioration of cutaneous photoageing by (0.3%) retinol. Int J Cosmet Sci. 2022;44(6):625–635. 35778881 10.1111/ics.12799 PMC9826105

[87] Diridollou S , Vienne MP , Alibert M , Aquilina C , Briant A , Dahan S , et al. Efficacy of topical 0.05% retinaldehyde in skin aging by ultrasound and rheological techniques. Dermatology. 1999;199(Suppl 1):37–41. 10473959 10.1159/000051377

[88] Rouvrais C , Baspeyras M , Mengeaud V , Rossi AB . Antiaging efficacy of a retinaldehyde‐based cream compared with glycolic acid peel sessions: a randomized controlled study. J Cosmet Dermatol. 2018;17(6):1136–1143. 30027612 10.1111/jocd.12511

[89] Kim H , Kim B , Kim H , Um S , Lee J , Ryoo H , et al. Synthesis and in vitro biological activity of retinyl retinoate, a novel hybrid retinoid derivative. Bioorg Med Chem. 2008;16(12):6387–6393. 18511283 10.1016/j.bmc.2008.05.005

[90] Kim H , Kim N , Jung S , Mun J , Kim J , Kim B , et al. Improvement in skin wrinkles from the use of photostable retinyl retinoate: a randomized controlled trial. Br J Dermatol. 2010;162(3):497–502. 19849696 10.1111/j.1365-2133.2009.09483.x

[91] Fu JJJ , Hillebrand GG , Raleigh P , Li J , Marmor MJ , Bertucci V , et al. A randomized, controlled comparative study of the wrinkle reduction benefits of a cosmetic niacinamide/peptide/retinyl propionate product regimen vs. a prescription 0.02% tretinoin product regimen. Br J Dermatol. 2010;162(3):647–654. 20374604 10.1111/j.1365-2133.2009.09436.x PMC2841824

[92] Bissett DL , Oblong JE , Berge CA . Niacinamide: a B vitamin that improves aging facial skin appearance. Dermatol Surg. 2005;31(7 Pt 2):860–865, discussion 5. 10.1111/j.1524-4725.2005.31732 16029679

[93] Liu M , Chen S , Zhang Z , Li H , Sun G , Yin N , et al. Anti‐ageing peptides and proteins for topical applications: a review. Pharm Dev Technol. 2022;27(1):108–125. 34957891 10.1080/10837450.2021.2023569

[94] Hawkins S , Adamus J , Chiang C‐y , Covell E , O'Leary J , Lee J‐m . Retinyl propionate and climbazole combination demonstrates clinical improvement to the appearance of hyperpigmentation and deep wrinkling with minimal irritation. Int J Cosmet Sci. 2017;39(6):589–599. 28733999 10.1111/ics.12412

[95] Wang Q , Hu F , Hu X , Xie Y , Du L , Ye R . The synergistic effect of retinyl propionate and hydroxypinacolone retinoate on skin aging. J Cosmet Dermatol. 2023;22:2040–2049. 36762391 10.1111/jocd.15662

[96] Farooq U , Mahmood T , Shahzad Y , Yousaf AM , Akhtar N . Comparative efficacy of two anti‐aging products containing retinyl palmitate in healthy human volunteers. J Cosmet Dermatol. 2018;17(3):454–460. 29363259 10.1111/jocd.12500

[97] Rawlings AV , Stephens TJ , Herndon JH , Miller M , Liu Y , Lombard K . The effect of a vitamin A palmitate and antioxidant‐containing oil‐based moisturizer on photodamaged skin of several body sites. J Cosmet Dermatol. 2013;12(1):25–35. 23438139 10.1111/jocd.12023

[98] Watson REB , Long SP , Bowden JJ , Bastrilles JY , Barton SP , Griffiths CEM . Repair of photoaged dermal matrix by topical application of a cosmetic ‘antiageing’ product. Br J Dermatol. 2008;158(3):472–477. 18070204 10.1111/j.1365-2133.2007.08364.x

[99] Kang S , Duell EA , Fisher GJ , Datta SC , Wang ZQ , Reddy AP , et al. Application of retinol to human skin in vivo induces epidermal hyperplasia and cellular retinoid binding proteins characteristic of retinoic acid but without measurable retinoic acid levels or irritation. J Invest Dermatol. 1995;105(4):549–556. 7561157 10.1111/1523-1747.ep12323445

[100] Lee DD , Stojadinovic O , Krzyzanowska A , Vouthounis C , Blumenberg M , Tomic‐Canic M . Retinoid‐responsive transcriptional changes in epidermal keratinocytes. J Cell Physiol. 2009;220(2):427–439. 19388012 10.1002/jcp.21784 PMC4386731

[101] Wolf JE Jr . Potential anti‐inflammatory effects of topical retinoids and retinoid analogues. Adv Ther. 2002;19(3):109–118. 12201351 10.1007/BF02850266

[102] Kim MY , Lee SE , Chang JY , Kim SC . Retinoid induces the degradation of Corneodesmosomes and downregulation of *Corneodesmosomal cadherins*: implications on the mechanism of retinoid‐induced desquamation. Ann Dermatol. 2011;23(4):439–447. 22148010 10.5021/ad.2011.23.4.439 PMC3229936

[103] Chapellier B , Mark M , Messaddeq N , Calleja C , Warot X , Brocard J , et al. Physiological and retinoid‐induced proliferations of epidermis basal keratinocytes are differently controlled. EMBO J. 2002;21(13):3402–3413. 12093741 10.1093/emboj/cdf331 PMC125394

[104] Eichner R , Kahn M , Capetola RJ , Gendimenico GJ , Mezick JA . Effects of topical retinoids on cytoskeletal proteins: implications for retinoid effects on epidermal differentiation. J Invest Dermatol. 1992;98(2):154–161. 1370674 10.1111/1523-1747.ep12555767

[105] Chen S , Ostrowski J , Whiting G , Roalsvig T , Hammer L , Currier SJ , et al. Retinoic acid receptor gamma mediates topical retinoid efficacy and irritation in animal models. J Invest Dermatol. 1995;104(5):779–783. 7738355 10.1111/1523-1747.ep12606988

[106] Elias PM , Fritsch PO , Lampe M , Williams ML , Brown BE , Nemanic M , et al. Retinoid effects on epidermal structure, differentiation, and permeability. Lab Invest. 1981;44(6):531–540. 6939940

[107] Thacher SM , Standeven AM , Athanikar J , Kopper S , Castilleja O , Escobar M , et al. Receptor specificity of retinoid‐induced epidermal hyperplasia: effect of RXR‐selective agonists and correlation with topical irritation. J Pharmacol Exp Ther. 1997;282(2):528–534. 9262312

[108] Ellis CN , Weiss JS , Hamilton TA , Headington JT , Zelickson AS , Voorhees JJ . Sustained improvement with prolonged topical tretinoin (retinoic acid) for photoaged skin. J Am Acad Dermatol. 1990;23(4 Pt 1):629–637. 2229490 10.1016/0190-9622(90)70265-j

[109] Goffin V , Henry F , Pierard‐Franchimont C , Pierard GE . Topical retinol and the stratum corneum response to an environmental threat. Skin Pharmacol. 1997;10(2):85–89. 9257377 10.1159/000211473

[110] Draelos ZD , Ertel KD , Berge CA . Facilitating facial retinization through barrier improvement. Cutis. 2006;78(4):275–281. 17121065

[111] Bernard FX , Pedretti N , Rosdy M , Deguercy A . Comparison of gene expression profiles in human keratinocyte mono‐layer cultures, reconstituted epidermis and normal human skin; transcriptional effects of retinoid treatments in reconstituted human epidermis. Exp Dermatol. 2002;11(1):59–74. 11952828 10.1034/j.1600-0625.2002.110107.x

[112] Jiang X , Teng M , Ji R , Zhang D , Zhang Z , Lv Y , et al. CD9 regulates keratinocyte differentiation and motility by recruiting E‐cadherin to the plasma membrane and activating the PI3K/Akt pathway. Biochim Biophys Acta Mol Cell Res. 2020;1867(2):118574. 31682865 10.1016/j.bbamcr.2019.118574

[113] Halai P , Kiss O , Wang R , Chien AL , Kang S , O'Connor C , et al. Retinoids in the treatment of photoageing: a histological study of topical retinoid efficacy in black skin. J Eur Acad Dermatol Venereol. 2024;38:1618–1627. 38682699 10.1111/jdv.20043

[114] Griffiths CE , Finkel LJ , Ditre CM , Hamilton TA , Ellis CN , Voorhees JJ . Topical tretinoin (retinoic acid) improves melasma. A vehicle‐controlled, clinical trial. Br J Dermatol. 1993;129(4):415–421. 8217756 10.1111/j.1365-2133.1993.tb03169.x

[115] Gano SE , Garcia RL . Topical tretinoin, hydroquinone, and betamethasone valerate in the therapy of melasma. Cutis. 1979;23(2):239–241. 421493

[116] Griffiths CE , Goldfarb MT , Finkel LJ , Roulia V , Bonawitz M , Hamilton TA , et al. Topical tretinoin (retinoic acid) treatment of hyperpigmented lesions associated with photoaging in Chinese and Japanese patients: a vehicle‐controlled trial. J Am Acad Dermatol. 1994;30(1):76–84. 8277035 10.1016/s0190-9622(94)70011-7

[117] Rafal ES , Griffiths CE , Ditre CM , Finkel LJ , Hamilton TA , Ellis CN , et al. Topical tretinoin (retinoic acid) treatment for liver spots associated with photodamage. N Engl J Med. 1992;326(6):368–374. 1729619 10.1056/NEJM199202063260603

[118] Sadick N , Edison BL , John G , Bohnert KL , Green B . An advanced, physician‐strength retinol peel improves signs of aging and acne across a range of skin types including melasma and skin of color. J Drugs Dermatol. 2019;18(9):918–923. 31524348

[119] Yoshimura K , Harii K , Aoyama T , Iga T . Experience with a strong bleaching treatment for skin hyperpigmentation in orientals. Plast Reconstr Surg. 2000;105(3):1097–1108; discussion 109–10. 10724272 10.1097/00006534-200003000-00040

[120] Yoshimura K , Harii K , Aoyama T , Shibuya F , Iga T . A new bleaching protocol for hyperpigmented skin lesions with a high concentration of all‐trans retinoic acid aqueous gel. Aesthetic Plast Surg. 1999;23(4):285–291. 10441721 10.1007/s002669900285

[121] Pathak MA , Fitzpatrick TB , Kraus EW . Usefulness of retinoic acid in the treatment of melasma. J Am Acad Dermatol. 1986;15(4 Pt 2):894–899. 3534025 10.1016/s0190-9622(86)70247-8

[122] Kligman AM , Willis I . A new formula for depigmenting human skin. Arch Dermatol. 1975;111(1):40–48. 1119822

[123] Yoshimura K , Tsukamoto K , Okazaki M , Virador VM , Lei TC , Suzuki Y , et al. Effects of all‐trans retinoic acid on melanogenesis in pigmented skin equivalents and monolayer culture of melanocytes. J Dermatol Sci. 2001;27(Suppl 1):S68–S75. 11514127 10.1016/s0923-1811(01)00116-5

[124] Welsh BM , Mason RS , Halliday GM . Topical all‐trans retinoic acid augments ultraviolet radiation‐induced increases in activated melanocyte numbers in mice. J Invest Dermatol. 1999;112(3):271–278. 10084301 10.1046/j.1523-1747.1999.00510.x

[125] Tancrede‐Bohin E , Baldeweck T , Brizion S , Decenciere E , Victorin S , Ngo B , et al. In vivo multiphoton imaging for non‐invasive time course assessment of retinoids effects on human skin. Skin Res Technol. 2020;26:794–803. 32713074 10.1111/srt.12877 PMC7754381

[126] Robert L , Robert AM , Renard G . Biological effects of hyaluronan in connective tissues, eye, skin, venous wall. Role in aging. Pathol Biol. 2010;58(3):187–198. 19932571 10.1016/j.patbio.2009.09.010

[127] Ghersetich I , Lotti T , Campanile G , Grappone C , Dini G . Hyaluronic acid in cutaneous intrinsic aging. Int J Dermatol. 1994;33(2):119–122. 8157393 10.1111/j.1365-4362.1994.tb01540.x

[128] Li W‐H , Wong H‐K , Serrano J , Randhawa M , Kaur S , Southall MD , et al. Topical stabilized retinol treatment induces the expression of HAS genes and HA production in human skin in vitro and in vivo. Arch Dermatol Res. 2017;309(4):275–283. 28247017 10.1007/s00403-017-1723-6

[129] Randolph RK , Simon M . Dermal fibroblasts actively metabolize retinoic acid but not retinol. J Invest Dermatol. 1998;111(3):478–484. 9740244 10.1046/j.1523-1747.1998.00307.x

[130] Haynes SL , Shuttleworth CA , Kielty CM . Keratinocytes express fibrillin and assemble microfibrils: implications for dermal matrix organization. Br J Dermatol. 1997;137(1):17–23. 9274620

[131] Varani J , Warner RL , Gharaee‐Kermani M , Phan SH , Kang S , Chung JH , et al. Vitamin A antagonizes decreased cell growth and elevated collagen‐degrading matrix metalloproteinases and stimulates collagen accumulation in naturally aged human skin. J Invest Dermatol. 2000;114(3):480–486. 10692106 10.1046/j.1523-1747.2000.00902.x

[132] Fisher GJ , Datta SC , Talwar HS , Wang ZQ , Varani J , Kang S , et al. Molecular basis of sun‐induced premature skin ageing and retinoid antagonism. Nature. 1996;379(6563):335–339. 8552187 10.1038/379335a0

[133] Sorg O , Antille C , Kaya G , Saurat JH . Retinoids in cosmeceuticals. Dermatol Ther. 2006;19(5):289–296. 17014484 10.1111/j.1529-8019.2006.00086.x

[134] Draelos ZD , Peterson RS . A double‐blind, comparative clinical study of newly formulated retinol serums vs tretinoin cream in escalating doses: a method for rapid retinization with minimized irritation. J Drugs Dermatol. 2020;19(6):625–631. 32574009 10.36849/JDD.2020.10.36849/JDD.2020.5085

[135] Kafi R , Kwak HS , Schumacher WE , Cho S , Hanft VN , Hamilton TA , et al. Improvement of naturally aged skin with vitamin A (retinol). Arch Dermatol. 2007;143(5):606–612. 17515510 10.1001/archderm.143.5.606

[136] Macdonald A , Fry L . Retinoic acid in the treatment of psoriasis. Br J Dermatol. 1972;86(5):524–527. 4556952 10.1111/j.1365-2133.1972.tb16108.x

[137] Callender VD , Baldwin H , Cook‐Bolden FE , Alexis AF , Stein Gold L , Guenin E . Effects of topical retinoids on acne and post‐inflammatory hyperpigmentation in patients with skin of color: a clinical review and implications for practice. Am J Clin Dermatol. 2022;23(1):69–81. 34751927 10.1007/s40257-021-00643-2 PMC8776661

[138] Langton AK , Alessi S , Hann M , Chien AL , Kang S , Griffiths CEM , et al. Aging in skin of color: disruption to elastic fiber organization is detrimental to skin's biomechanical function. J Invest Dermatol. 2019;139(4):779–788. 30404021 10.1016/j.jid.2018.10.026

